# Improvements in Glycemic Control With a Digital Diabetes Logbook: Secondary Analysis of a Randomized Controlled Trial Enriched by Observational, Real-World Data

**DOI:** 10.2196/68933

**Published:** 2025-06-30

**Authors:** Dominic Ehrmann, Bernhard Ruch, Michael Mitter, Johanna Kober, Norbert Hermanns, Vanessa Schäfer, Bernhard Kulzer, Stephan Silbermann

**Affiliations:** 1 Research Institute of the Diabetes Academy Mergentheim Forschungsinstitut der Diabetes-Akademie Bad Mergentheim Bad Mergentheim Germany; 2 University of Bamberg Department of Clinical Psychology and Psychotherapy Bamberg Germany; 3 mySugr GmbH Vienna Austria; 4 Roche Diabetes Care GmbH Mannheim Germany; 5 Roche Diabetes Care Deutschland GmbH Mannheim Germany

**Keywords:** randomized controlled trial, real-world data, glycemic control, digital health app, type 1 diabetes, type 2 diabetes

## Abstract

**Background:**

The treatment of diabetes requires substantial self-management. Digital tools can help reduce the burden of self-management and may improve glycemic control.

**Objective:**

This study aims to determine whether the use of a digital diabetes logbook increased the likelihood of achieving optimal glycemic control (glycated hemoglobin [HbA_1c_] ≤6.5%) after 3 months, based on a secondary analysis of randomized controlled trial (RCT) data. A secondary objective was to evaluate the long-term impact of the logbook on mean blood glucose levels over 3 and 12 months using observational, real-world data (RWD).

**Methods:**

Data from 342 participants with type 1 or type 2 diabetes enrolled in the mySugr PRO-RCT were analyzed. A robust logistic regression was performed to examine the likelihood of achieving optimal glycemic control, defined as an HbA1c value ≤6.5% at the 3-month follow-up. The dependent variable was the dichotomous outcome indicating whether this threshold was met. The primary independent variable was group allocation, with baseline HbA1c included as a covariate. For the analysis of RWD, a total of 2861 participants with type 1 or type 2 diabetes were identified using propensity score matching to align their characteristics with those of the RCT participants closely. One-sample t tests were conducted to analyze changes in mean blood glucose separately for each diabetes type, from baseline to 3 months of app use, and from baseline to 12 months of app use (in a subcohort of 1176 participants).

**Results:**

The RCT data showed that the likelihood of achieving optimal glycemic control was nearly doubled in the intervention group compared with the control group (odds ratio 2.24, 95% CI 1.12-4.47; *P*=.02). RWD indicated that mean blood glucose levels significantly improved over 3 months of app use in both groups (type 1: –16.3 mg/dL; 95% CI –20.6 to –12.4; *P*<.001 and type 2: –27.3 mg/dL, 95% CI –28.7 to –25.9; *P*<.001). Participants with an estimated HbA_1c_>8.5% at baseline (before app use) showed the greatest reductions in mean blood glucose (type 1: –82.2 mg/dL; 95% CI –102.0 to –61.8; *P*<.001; type 2: –104.6 mg/dL, 95% CI –109.1 to –100.3; *P*<.001). Long-term analyses revealed a sustained reduction in mean blood glucose over a 12-month period, with a mean decrease of –19.8 mg/dL (95% CI –21.8 to –17.9; *P*<.001) after 12 months of app use in the total RWD sample.

**Conclusions:**

The secondary analysis of the RCT demonstrated a significant increase in the likelihood of achieving optimal glycemic control after 3 months of using the mySugr logbook. This finding was supported by observational, real-world data, which showed significant reductions in mean blood glucose after 3 and 12 months of app use—particularly among individuals with elevated baseline HbA1c levels.

**Trial Registration:**

German Clinical Trials Register DRKS00022923; https://drks.de/search/en/trial/DRKS00022923/details

## Introduction

Digital tools for diabetes management are becoming increasingly popular [1]. Several meta-analyses have examined the efficacy of digital, mobile, or eHealth interventions, particularly for people with type 2 diabetes [1-6]. Regarding glycemic control, the meta-analyses by Kerr et al [2], Romadlon et al [3], Moschonis et al [1], and Zhang et al [4] all demonstrated significant improvements in glycated hemoglobin (HbA_1c_) with digital interventions compared with control groups. In the Bayesian meta-analysis by Zhang et al [4], which included 88 studies and 13,972 people with type 2 diabetes, a mean HbA_1c_ reduction of –0.45% (95% CI –0.61 to –0.30) was found for smartphone apps [4]. This is corroborated by Moschonis et al [1], who found a reduction of –0.42% (95% CI –0.63 to –0.20) for smartphone apps, and by Kerr et al [2], who reported a respective reduction of –0.31% (95% CI –0.45 to –0.16) for all types of digital interventions.

Besides glycemic control, meta-analytic evidence also suggests that technology-based or digital interventions can improve psychosocial well-being by reducing diabetes distress [5,6]. Thus, there is evidence that digital tools can effectively support people with diabetes in managing their therapy.

To fully understand the efficacy of a digital intervention, data from randomized controlled trials (RCTs) should be complemented by real-world data (RWD) [7,8]. RWD generally offer higher external validity due to the greater heterogeneity of the included sample, as strict inclusion and exclusion criteria—common in RCTs—are typically absent. Furthermore, observation periods in RWD are often longer than in RCTs, allowing for the assessment of effects over an extended time frame.

For many digital interventions, although efficacy is tested in an RCT [1-6], subsequent evidence for effectiveness using RWD is often lacking.

One of the most widely used digital tools for people with diabetes is the digital diabetes logbook *mySugr.* To obtain regulatory approval and build an evidence base for this specific app, an RCT was conducted to demonstrate its efficacy [9]. In that trial, the digital logbook showed significant improvements in diabetes distress [9], an important indicator of psychosocial health and a common mental health concern among people with diabetes [10,11]. Furthermore, incidence rate ratios of severe hypoglycemic (<54 mg/dL) and hyperglycemic (>250 mg/dL) glucose measurements were significantly reduced in the intervention group, indicating the beneficial effects of the mySugr app on glycemic control and the safer management of diabetes therapy. However, the effect of mySugr on glycemic end points (eg, HbA_1c_) was not conclusive and requires further evaluation. The RCT included people with type 1 and type 2 diabetes, as well as women with gestational diabetes mellitus (GDM). Consequently, the overall baseline HbA_1c_ of 7.1% indicated optimal glycemic control, leaving little room for improvement—or, in the case of women with GDM, no clinical indication to lower HbA_1c_ values [9]. Therefore, secondary analyses and data from real-world users were used to build on the results of the RCT.

To further investigate the potential effects of the mySugr app on glycemic control, we conducted secondary analyses using the RCT data and an additional effectiveness analysis with RWD. Specifically, our analysis had 2 aims: (1) to assess whether the proportion of people with type 1 and type 2 diabetes who achieved an HbA_1c_ value below 6.5% [12] at follow-up was greater in the intervention arm of the RCT compared with the control group and (2) to evaluate the long-term effects on glycemic control in a real-world sample of existing mySugr users.

## Methods

### Randomized Controlled Trial

#### Study Design

The study design and main outcomes of the RCT have been published previously [9,13]. In brief, the trial (German Clinical Trials Register: DRKS00022923) was a randomized, controlled, open-label, parallel-group study with a 2:1 allocation ratio favoring the intervention group. The 3-month follow-up compared the intervention arm—using mySugr for 3 months—with a control group without a digital health intervention. The key inclusion criteria were a diagnosis of type 1 diabetes, type 2 diabetes, or GDM; regular daily self-monitoring of blood glucose; age≥16 years; and a most recent HbA_1c_ value <12% (107.6 mmol/mol). The key exclusion criterion was the use of a continuous glucose monitoring (CGM) system. The RCT was conducted in Germany across 41 secondary care practices. For this secondary analysis, only data from individuals with type 1 or type 2 diabetes were used. Additionally, the analysis focused exclusively on HbA_1c_ as the marker of glycemic control. This secondary analysis was not prespecified.

#### Digital Diabetes Logbook

The app was developed to support individuals with diabetes in the daily management of their condition. It integrates a range of diabetes-related data, including glucose levels, estimated HbA_1c_ (eHbA_1c_) values, and insulin intake, as well as entries on diet, weight, blood pressure, activity levels, and stress. These data can be shared with the diabetes care team before appointments. The app provides users with an overview of their diabetes data through easy-to-interpret reports that use traffic light colors to highlight areas for attention and suggest opportunities for improvement. However, it should be noted that the app does not offer recommendations for therapy adjustments. In addition, it incorporates psychological features such as motivational challenges and positive feedback. A more detailed description of the app is available elsewhere [9].

#### Assessment of Glycemic Control

HbA_1c_ was used as the marker of glycemic control, assessed at baseline and at the 3-month follow-up visit. HbA_1c_ levels were measured using venous blood samples.

#### Statistical Analysis

The difference in the proportion of participants achieving an HbA_1c_ value ≤6.5%, based on the treatment algorithm of the American Association of Clinical Endocrinologists (AACE) [12], at the 3-month follow-up was analyzed using robust logistic regression. The dependent variable was a dichotomized indicator of whether a participant achieved an HbA_1c_ value ≤6.5%. The independent variable of interest was group allocation, and the analysis was adjusted for baseline HbA_1c_. Only participants with type 1 or type 2 diabetes were included in this analysis, as none of the women with GDM had baseline HbA_1c_ values above 6.5%. The analysis was conducted for both the intention-to-treat population (ie, all randomized participants) and the per-protocol population. The per-protocol population was defined as participants who met all eligibility criteria, completed the follow-up visit within 42-137 days (ie, within 50% of the planned 84-day study period), used the intervention app on at least 10% of study days (intervention group), did not use any digital diabetes diary (control group), and did not use CGM during the study period—as prespecified in the RCT analysis plan [9]. Missing HbA_1c_ data in the RCT were imputed using multiple imputation (see [9,13]).

#### Ethical Considerations

Ethical approval was obtained from the State Chamber of Physicians of Baden-Wuerttemberg (approval number F-2020-121) as the primary vote, as well as from the 13 local State Chambers of the participating study sites. All participants provided written informed consent, which also covered the use of their data for both primary and secondary analyses. Therefore, no additional consent for secondary analyses was required. All RCT data were handled using participation codes (pseudonymization). The list linking participant names to these codes was securely stored at the study sites and destroyed after the study concluded. As a result, only anonymous, deidentified study data were used for all analyses. Participants received a compensation of €50 (US $57.78) for participation in the RCT, which was approved and deemed appropriate by the ethics committee.

### Observational, Real-World Data

#### Study Design

RWD was drawn from a user database of existing mySugr users. For this analysis, only users with type 1 or type 2 diabetes from Germany were included, as there is no clinical indication to lower HbA_1c_ in women with GDM. To align with the RCT’s exclusion criteria, only users who were not using a CGM were selected. To enable analysis of glucose trajectories and comparisons of glucose levels before and after app use, users were required to have uploaded glucose data before initiating app use (defined as baseline, or month – 1) and to have logged at least one blood glucose measurement per month. Glucose data were extracted for 2 cohorts: (1) from baseline (month – 1) up to month 3 of app use, and (2) from baseline up to month 12. The end point of interest was eHbA_1c_ at various time points, used as a marker of glycemic control.

#### Propensity Score Matching

Propensity score matching was used to select users from the existing user database to enhance the comparability of the RWD with the intervention group of the RCT. This approach was intended to mitigate the lack of randomization and minimize confounding [7]. The following variables were used for direct matching to the intervention group: age (in 10-year intervals), diabetes type, and initial eHbA_1c_ (categorized in 1% steps from 5% to 10%). In addition, propensity scores were calculated within these directly matched groups based on app usage and other demographic variables. The following covariates were included in the propensity score model: years since diagnosis, self-reported gender, self-reported therapy type, metformin use, number of blood glucose logs, number and the average value of carbohydrate logs, total steps logged, number and the average value of BMI logs, number and the average value of blood pressure logs, number and total duration of exercise logs, number and the average value of basal insulin injections, number and the average value of bolus insulin injections, number of challenges completed in the mySugr app, number of tags and notes added to log entries, number of meal images uploaded, and use of the mySugr bolus calculator. All features were extracted from the first 30 days of mySugr use for both the RCT population and the existing user database. Categorical features were one-hot encoded. To address the class imbalance, the existing user database was randomly undersampled, retaining 20% of the original data. Missing values were imputed using a K-nearest neighbors imputer with the 2 closest neighbors. The data were then standardized, and propensity scores were calculated using a logistic regression model, with membership in the RCT population as the treatment indicator. Matching within the directly matched strata was performed by selecting the 50 closest neighbors for each treated individual based on the logit-transformed output of the classifier, allowing replacement and applying a maximum allowable distance of 0.4. Matching success was assessed using Cohen *d* values for all included variables.

#### Digital Diabetes Logbook

The same app as in the RCT was used in the observational, real-world study.

#### Assessment of Glycemic Control

Glucose data uploaded from blood glucose meters into the mySugr app were used to calculate the mean glucose for each month over a 12-month period. The eHbA_1c_ before app use was calculated using the formula by Nathan et al [14].

#### Statistical Analysis

Changes in mean glucose at month 3 relative to the month before app use were analyzed using a paired, 2-sided *t* test to assess whether the change differed significantly from 0. Additionally, a 1-sample Wilcoxon signed rank test was conducted to confirm that the rejection of the null hypothesis was not driven by violations of the *t* test assumptions. All *P* values were adjusted using the Bonferroni correction to account for multiple testing. To analyze long-term changes, the difference in mean glucose from before app use to month 12 was assessed using the same procedure. Missing data were not imputed. In addition, changes in mean glucose were analyzed separately for people with type 1 and type 2 diabetes, stratified by eHbA_1c_ values before app use (≤7.5%, 7.6%-8.0%, 8.1%-8.5%, and >8.5%).

*P* values <.05 were considered statistically significant. All statistical analyses were conducted using R version 4.2.3 (R Foundation; package robustbase for robust logistic regression) and Python version 3.11.4 (Python Foundation), with the following packages: statsmodels version 0.14.0 for all statistical tests, pandas version 2.1.1 for data transformations, and scikit-learn version 1.3.0 for propensity scoring and feature preprocessing.

#### Ethical Considerations

As part of the onboarding process, mySugr app users can consent to their data being used for research purposes. Only data from users who provided such consent were included in the analyses. The mySugr app adheres to rigorous data privacy and protection standards [15], and only anonymous, deidentified data were used for analysis. Users did not receive any compensation. Given these considerations, ethics committee approval for this specific study was waived (WCG institutional review board tracking number 20251379).

## Results

### Randomized Controlled Trial

#### Participants

For the RCT data, a total of 342 participants with type 1 or type 2 diabetes were included in the intention-to-treat population, with 225 randomized to the intervention group and 117 to the control group. The per-protocol population consisted of 285 participants with type 1 or type 2 diabetes who completed the RCT in accordance with the study protocol. As shown in Table 1, the majority of participants in the RCT had type 2 diabetes and were receiving insulin therapy.

**Table 1:** Baseline characteristics of participants from the randomized control trials and real-world cohort.

Characteristics		Randomized controlled trial			Real-world cohort (3 months)		Real-world cohort (12 months)
		Control group (n=117)	Intervention group (n=225)	Existing app users (n=2861)		Existing app users (n=1176)	
Age (years), mean (SD)		57.3 (14.2)	55.7 (12.7)	56.8 (9.7)		58.9 (9.0)	
**Gender^a^, n (%)**							
	Female	47 (40.2)	85 (37.8)	452 (15.8)		187 (15.9)	
	Male	70 (59.8)	140 (62.2)	1047 (36.6)		490 (41.7)	
	Diverse	0 (0)	0 (0)	0 (0)		0 (0)	
BMI (kg/m^2^), mean (SD)		31.0 (6.3)	33.1 (7.3)	32.6 (6.1)		32.7 (4.8)	
**Type of diabetes, n (%)**							
	Type 1 diabetes	16 (13.7)	37 (16.4)	348 (12.2)		147 (12.5)	
	Type 2 diabetes	101 (86.3)	188 (83.6)	2513 (87.8)		1029 (87.5)	
Diabetes duration (years), mean (SD)		12.4 (12.4)	11.2 (10.1)	6.5 (6.9)		8.2 (8.0)	
**Diabetes therapy, n (%)**							
	Lifestyle	42 (35.9)	76 (33.8)	N/A^b^		N/A^b^	
	Oral antidiabetic medication	66 (56.4)	146 (64.9)	N/A^b^		N/A^b^	
	Incretins	33 (28.2)	53 (23.6)	N/A^b^		N/A^b^	
	Insulin	85 (72.6)	158 (70.2)	N/A^b^		N/A^b^	
	Insulin pump	2 (1.7)	3 (1.3)	N/A^b^		N/A^b^	
Number of long-term complications^c^, mean (SD)		0.93 (1.28)	0.78 (1.06)	N/A^b^		N/A^b^	
**HbA_1c_^d,e^, mean (SD)**							
	%	7.5 (1.3)	7.5 (1.3)	7.4 (2.0)		7.4 (1.7)	
	mmol/mol	58 (14.2)	58 (14.2)	57 (21.9)		57 (19.0)	
**HbA_1c_ categories^d^, n (%)**							
	≤7.5%	69 (59.0)	136 (60.4)	1868 (65.3)		798 (67.9)	
	7.6%-8.0%	11 (9.4)	27 (12.0)	220 (7.7)		88 (7.5)	
	8.1%-8.5%	13 (11.1)	21 (9.3)	196 (6.9)		71 (6.0)	
	>8.5%	23 (19.7)	40 (17.8)	577 (20.2)		219 (18.6)	

^a^Gender is voluntary information for the mySugr app users and thus not all users provide this information.

^b^N/A: not applicable (information is not available from mySugr user data).

^c^List of long-term complications: retinopathy, neuropathy, nephropathy, peripheral arterial occlusive disease, coronary heart disease, myocardial infarction, and stroke.

^d^Laboratory-measured HbA_1c_ for the RCT and eHbA_1c_ for the real-world cohort.

^e^HbA_1c_: glycated hemoglobin.

#### Improvement in Glycemic Control in the RCT

In the intention-to-treat population, 74 out of 225 (32.9%) participants in the intervention group achieved an HbA_1c_ value ≤6.5% 3 months after baseline, compared with 31 out of 117 (26.5%) participants in the control group (Multimedia Appendix 2). When controlling for baseline HbA_1c_ and accounting for the nonnormal distribution of HbA_1c_ values, the likelihood of achieving an HbA_1c_ value ≤6.5% at 3 months was nearly twice as high in the intervention group compared with the control group (odds ratio 2.24, 95% CI 1.12-4.47; *P*=.02). In the per-protocol population, 59 out of 183 (32.2%) participants in the intervention group achieved an HbA_1c_ value ≤6.5% at 3 months, compared with 26 out of 102 (25.5%) participants in the control group. Similarly, the likelihood of achieving an HbA_1c_ value ≤6.5% was 2.26 times higher in the intervention group than in the control group (odds ratio 2.26, 95% CI 1.09-4.71; *P*=.03), when controlling for baseline HbA_1c_ and accounting for the nonnormal distribution.

### Observational, Real-World Data

#### Participants

For the RWD analysis, a total of 2861 users with at least three months of glucose data were selected, including 348 with type 1 diabetes and 2513 with type 2 diabetes. Propensity score matching was successful, as all demographic variables were comparable between the RCT participants and the RWD cohort (Table 1). Figure S1 in Multimedia Appendix 1 also illustrates the effectiveness of the propensity score matching. At baseline, the majority of individuals with type 1 (223/348, 64.1%) and type 2 diabetes (1645/2513, 65.5%) had an eHbA_1c_≤7.5%. Data from 147 users with type 1 diabetes and 1029 users with type 2 diabetes were sufficient for inclusion in the 12-month app use analysis.

#### Short-Term Glucose Trajectories After App Use in the Real-World Cohort

Three months after baseline (with month – 1 indicating the month before app use), 1670 out of 2861 (58.37%) users achieved an eHbA_1c_ value ≤6.5%. Among them, 704 (42.16%) users showed an improvement in eHbA_1c_ compared with the baseline. By contrast, only 176 out of 2861 (6.15%) users experienced a deterioration, with an eHbA_1c_>6.5% at 3 months despite having a baseline eHbA_1c_≤6.5%.

**Figure 1:**
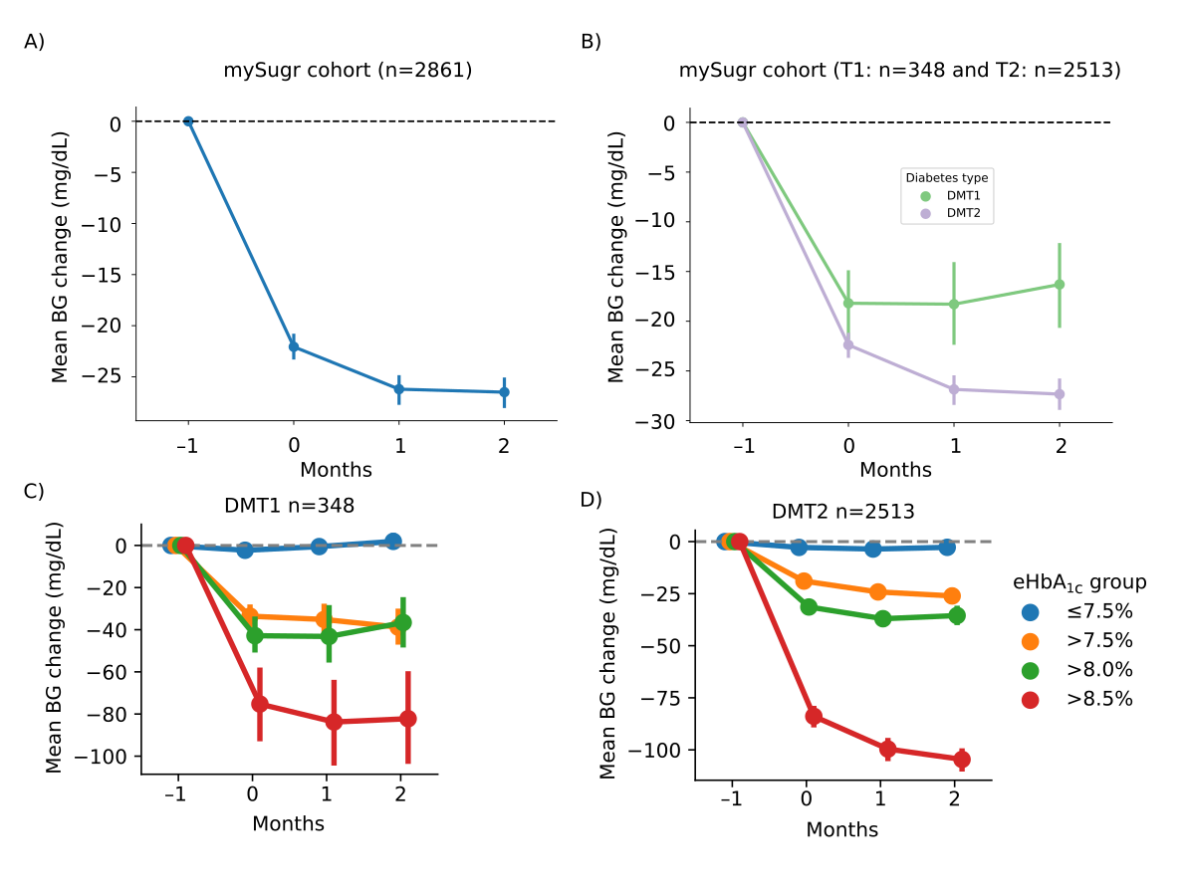
Reduction in mean blood glucose (BG) levels before and during 3 months of app use (A) in the total sample, (B) in people with diabetes mellitus type 1 (DMT1) and diabetes mellitus type 2 (DMT2), and stratified by baseline estimated glycated hemoglobin (eHbA1c) in (C) DMT1 and (D) DMT2.

Overall, mean glucose levels decreased from 167 mg/dL at baseline (month before app use) to 140.5 mg/dL 3 months after app use began (*P*<.001; Table 2). This corresponds to a mean reduction of 26.5 mg/dL in the total RWD cohort (Figure 1A, Table 2). Notably, the majority of this reduction occurred during the first month of app use (–22.1 mg/dL) and remained stable thereafter (Figure 1A, Table S1 in Multimedia Appendix 1). Similar glucose trajectories were observed in both people with type 1 (*P*<.001; Table 2, Figure 1B) and type 2 diabetes (*P*<.001; Table 2, Figure 1B), with mean reductions of 16.3 mg/dL (95% CI –20.6 to –12.4) and 27.3 mg/dL (95% CI –28.7 to –25.9), respectively, at 3 months postbaseline (Table S1 in Multimedia Appendix 1). Reductions were most pronounced in individuals with a baseline eHbA_1c_>8.5%, both in those with type 1 diabetes (–82.2 mg/dL; 95% CI –102.0 to –61.8; Figure 1C, Table S2 in Multimedia Appendix 1) and type 2 diabetes (–104.6 mg/dL; 95% CI –109.1 to –100.3; Figure 1D, Table S3 in Multimedia Appendix 1). Significant reductions in mean glucose over the 3 months following baseline were also observed among users with baseline eHbA_1c_>7.5% (type 1: *P*<.001; type 2: *P*<.001) and >8.0% (type 1: *P*<.001; type 2: *P*<.001). Reductions were also significant (*P*<.001) among individuals with type 2 diabetes and baseline eHbA_1c_≤7.5%. However, no change was observed in individuals with type 1 diabetes and baseline eHbA_1c_≤7.5%. Figure S2A-C in Multimedia Appendix 1 illustrates the corresponding mean blood glucose levels before and during the 3 months of app use. Tables S1-S3 in Multimedia Appendix 1 present monthly reductions in mean glucose across the full sample, as well as separately for people with type 1 and type 2 diabetes. Results from the Wilcoxon signed rank test were consistent with those from the paired *t* test (data not shown).

**Table 2:** Glucose values (in mg/dL) at baseline and 3 months after app use.

Sample				Baseline, mean (SD)		3 months of app use, mean (SD)		Mean (SD) change		Test statistic^a,b^		*P* value^c^		
All (N=2861)				167.0 (56.3)		140.5 (31.4)		–26.5 (58.1)		–39.8		<.001		
Type 1 diabetes (n=348)				157.6 (50.6)		141.3 (39.4)		–16.3 (49.7)		–7.9		<.001		
Type 2 diabetes (n=2513)				167.8 (56.7)		140.4 (30.7)		–27.3 (58.7)		–39.1		<.001		
**eHbA_1c_^d^ categories**														
	**Type 1 diabetes**													
		≤7.5%	132.7 (19.9)		134.6 (25.7)		1.9 (25.1)		1.5		>.99^e^			
		7.6%-8.0%	175.3 (3.5)		136.6 (30.7)		–38.8 (30.5)		–10.2		<.001			
		8.1%-8.5%	189.2 (3.6)		152.6 (35.9)		–36.6 (36.3)		–6.7		<.001			
		>8.5%	256.4 (63.0)		174.2 (75.7)		–82.2 (87.7)		–8.0		<.001			
	**Type 2 diabetes**													
		≤7.5%	137.0 (17.5)		134.3 (23.3)		–2.8 (22.3)		–8.4		<.001			
		7.6%-8.0%	174.1 (4.2)		148.0 (30.4)		–26.1 (29.9)		–20.0		<.001			
		8.1%-8.5%	189.7 (3.9)		154.2 (37.7)		–35.5 (38.5)		–21.7		<.001			
		>8.5%	256.8 (62.7)		152.2 (41.4)		–104.6 (82.5)		–47.5		<.001			

^a^As the cohort was created using propensity scoring with resampling, the number of unique users is smaller than the number of degrees of freedom in the data set that was used for the tests.

^b^Based on a paired *t* test.

^c^*P* value adjusted for multiple testing using the Bonferroni method (22 tests).

^d^eHbA_1c_: estimated glycated hemoglobin.

^e^For reporting, instead of lowering the test value using the Bonferroni method, we multiplied *P* values by the number of tests performed. Thus, values above 1 are possible and we capped the value at 1.

#### Long-Term Glucose Trajectories After App Use in the Real-World Cohort

**Figure 2:**
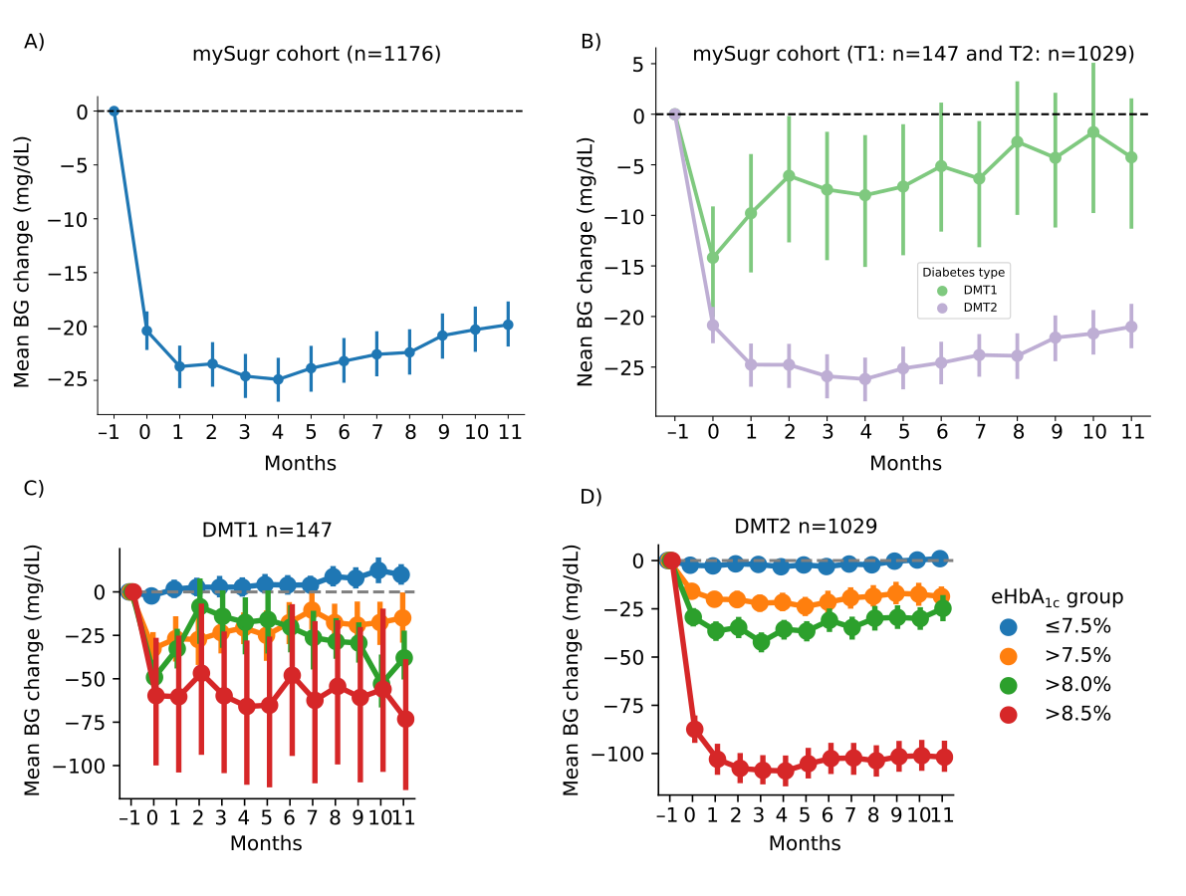
Reduction in mean blood glucose (BG) levels before and during 12 months of app use (A) in the total sample, (B) in people with diabetes mellitus type 1 (DMT1) and diabetes mellitus type 2 (DMT2), and stratified by baseline estimated glycated hemoglobin (eHbA1c) in (C) DMT1 and (D) DMT2.

In the total sample, mean glucose levels significantly decreased after 12 months of app use compared with baseline (*P*<.001; Figure 2A). In the final month, mean glucose was reduced by 19.8 mg/dL, from 165.9 mg/dL to 146.1 mg/dL (Table 3, Figure 2A). Among individuals with type 1 diabetes, an initial reduction of 14.2 mg/dL (95% CI –19.8 to –9.3 mg/dL) was observed after 1 month of app use. However, this effect diminished over time, with a net reduction of only 4.3 mg/dL after 12 months (Table 3, Figure 2B). By contrast, individuals with type 2 diabetes experienced a sustained and significant reduction in mean glucose of 21.0 mg/dL at 12 months compared with baseline (*P*<.001; Table 3, Figure 2B). The greatest improvements were observed in users with baseline eHbA_1c_>8.5%, with reductions of 73.2 mg/dL in type 1 diabetes (*P*=.02; Table 3, Figure 2C) and 101.9 mg/dL in type 2 diabetes (*P*<.001; Table 3, Figure 2D). Figure S3A-C in Multimedia Appendix 1 displays the corresponding mean blood glucose levels before and during the 12 months of app use.

**Table 3:** Glucose (in mg/dL) values at baseline and 12 months after app use.

Sample				Baseline, mean (SD)		12 months of app use, mean (SD)		Mean (SD) change		Test statistic^a,b^		*P* value^c^		
All (N=1176)				165.9 (53.6)		146.1 (32.9)		–19.8 (57.3)		–19.6		<.001		
Type 1 diabetes (n=147)				156.4 (53.1)		152.1 (36.1)		–4.3 (49.8)		–1.3		.44		
Type 2 diabetes (n=1029)				166.6 (53.6)		145.6 (32.6)		–21.0 (57.7)		–19.9		<.001		
**eHbA_1c_^d^categories**														
	**Type 1 diabetes**													
		≤7.5%	134.7 (19.9)		144.6 (27.2)		9.9 (30.6)		4.2		.001			
		7.6%-8.0%	175.1 (3.7)		160.1 (29.4)		–15.0 (30.0)		–2.3		.73			
		8.1%-8.5%	189.5 (2.6)		151.5 (22.0)		–38.0 (24.2)		–5.9		.001			
		>8.5%	270.6 (81.5)		197.4 (61.1)		–73.2 (95.6)		–3.8		.02			
	**Type 2 diabetes**													
		≤7.5%	138.4 (16.9)		139.4 (25.4)		1.0 (25.3)		1.7		>.99^e^			
		7.6%-8.0%	174.3 (4.1)		155.7 (30.9)		–18.6 (30.7)		–8.9		<.001			
		8.1%-8.5%	189.3 (3.0)		164.5 (42.0)		–24.8 (42.2)		–9.2		<.001			
		>8.5%	258.1 (57.7)		156.2 (43.3)		–101.9 (81.4)		–29.0		<.001			

^a^As the cohort was created using propensity scoring with resampling, the number of unique users is smaller than the number of degrees of freedom in the data set that was used for the tests.

^b^Based on a paired 1-sample *t* test.

^c^*P* value adjusted for multiple testing using the Bonferroni method (22 tests).

^d^eHbA_1c_: estimated glycated hemoglobin.

^e^For reporting, instead of lowering the test value using the Bonferroni method, we multiplied *P* values by the number of tests performed. Thus, values above 1 are possible and we capped the value at 1.

For the cohort with 12 months of data available, monthly reductions in mean blood glucose for the different subgroups are presented in Tables S4-S6 in Multimedia Appendix 1. Among individuals with type 1 diabetes, the greatest numerical reduction in mean glucose compared with baseline was observed at month 1 (Table S4 in Multimedia Appendix 1). For individuals with type 2 diabetes, the largest reduction occurred at month 4 (Table S4 in Multimedia Appendix 1). Similar patterns were confirmed using the Wilcoxon signed rank test (data not shown).

## Discussion

### Principal Findings

This secondary analysis of the RCT demonstrated that use of the mySugr app was associated with a 2-fold increase in the likelihood of achieving HbA_1c_ values ≤6.5% 3 months after baseline, compared with a control group without app use. Additionally, analyses of RWD from existing mySugr users—matched to the RCT intervention group—supported and extended the findings from the RCT. Compared with the month before app use, users significantly reduced their mean blood glucose levels by approximately 20 mg/dL during the first 3 months of app use. Notably, these reductions were observed as early as the first month and were sustained over a 12-month period, with reductions at month 12 remaining around 20 mg/dL. This suggests a potential long-term benefit of using a digital logbook. A mean glucose reduction of 20 mg/dL corresponds to an eHbA_1c_ decrease of approximately 0.5%-0.7% [14,16]. The observed effect size is clinically meaningful, exceeding the noninferiority margin of 0.3%-0.4% established by both the US Food and Drug Administration [17] and the European Medicines Agency [18].

In addition, 3 months after initiating app use, mean glucose levels decreased to 140 mg/dL, corresponding to an eHbA_1c_ of 6.5% [14,16]. According to the AACE, this reflects near-normal glycemic levels [12], suggesting that the use of the digital diabetes logbook helped users achieve clinically desirable glucose control within a short period. At 12 months, mean glucose levels remained improved, although a slight increase was observed compared with month 3, corresponding to an eHbA_1c_ of 6.7%.

Taken together, the comparison of RWD with results from the RCT provides additional evidence that the use of a digital logbook is associated with improvements in glycemic control.

### Comparison With Prior Work

These results should be interpreted in the context of the safety analysis published alongside the main results of the RCT [9]. Throughout the RCT, the incidence rate ratio of severe hypoglycemic self-monitored blood glucose values <54 mg/dL was reduced by 25% in the intervention group compared with the control group (incidence rate ratio 0.75, 95% CI 0.57-0.99; *P*=.048) [9]. Taken together, this suggests that participants using the digital logbook were more likely to achieve an optimal HbA_1c_ level while simultaneously experiencing a lower risk of hypoglycemic episodes compared with those not using the app.

The estimated reduction in HbA_1c_ observed in the RWD cohort—approximately 0.5%-0.7%—aligns well with reductions reported in several meta-analyses evaluating smartphone-based digital interventions [1-4]. This supports the potential of digital health apps to improve glycemic management. Notably, the meta-analysis by Kerr et al [2] also provides evidence that digital interventions for self-management of type 2 diabetes can significantly improve HbA_1c_ even without the use of CGM. This is particularly relevant, as the use of CGM was an exclusion criterion in this analysis.

Over the 3- and 12-month periods of app use, reductions in mean glucose levels were greater among individuals with type 2 diabetes compared with those with type 1 diabetes. This observation is consistent with findings from the systematic review by Stevens et al [19], which reported smaller HbA_1c_ improvements in individuals with type 1 diabetes than those with type 2 diabetes. A possible explanation may lie in the greater complexity of type 1 diabetes management, which is characterized by higher glycemic variability compared with type 2 diabetes [20-22]. As expected, the most substantial reductions were observed in individuals with baseline eHbA_1c_ values >8.5%, regardless of diabetes type. For instance, people with type 2 diabetes and a baseline eHbA_1c_>8.5% experienced a reduction in mean blood glucose of approximately 100 mg/dL compared with the month before app use, corresponding to an eHbA_1c_ decrease of about 3.48% [14,16]. These findings underscore the clinical relevance of using a digital logbook, particularly for individuals with diabetes who are not meeting glycemic targets. Evidence indicates that a substantial proportion of people with both type 1 and type 2 diabetes continue to struggle with achieving recommended glycemic control [23,24]. For this population, a relatively low-intensity digital intervention—such as a digital logbook—may offer a practical and scalable option to support more effective diabetes self-management.

### Strengths and Limitations

Several limitations must be considered when interpreting these results. First, the secondary analysis of the RCT data was not prespecified, which may increase the risk of type I errors. Second, while diabetes distress was the primary outcome of the RCT, corresponding data were not available in the RWD, as this variable is not routinely collected from mySugr users. Consequently, real-world effects on diabetes distress could not be assessed. Third, both the RCT and RWD populations consisted of highly engaged users of the mySugr app who did not use CGM systems. This may indicate a selective population with higher digital affinity, which limits the generalizability of the findings. Lastly, HbA_1c_ had to be estimated from blood glucose values, as laboratory-assessed values were unavailable in the RWD. In addition, participants followed different therapy regimens, which may have affected the comparability of blood glucose measurements. For some users, only fasting glucose values were uploaded, whereas others recorded both fasting and postprandial values. This variability complicates between-participant comparisons. However, because changes in glucose levels were assessed within individuals over time, each participant effectively served as their own control, mitigating the impact of interindividual differences in measurement practices.

A key strength of this analysis is the matching of a real-world population with participants from an RCT, thereby increasing the external validity of the RCT findings. The results also provide a realistic estimate of the potential effects of the app when used consistently in everyday settings. Furthermore, the RWD extended the insights from the RCT by offering evidence of sustained effects over a 12-month period, beyond the initial 3-month time frame evaluated in the RCT.

### Conclusions and Future Directions

In conclusion, the RWD provide additional evidence supporting the potential glycemic benefits of using a digital diabetes logbook. While the RCT did not show a significant reduction in HbA_1c_—likely due to the already low baseline HbA_1c_ of 7.1% and the inclusion of women with GDM [9]—the RWD analyses demonstrated that mean blood glucose levels can be substantially reduced through app use, with improvements maintained for up to 12 months. Notably, individuals with type 2 diabetes and those not achieving glycemic targets exhibited the greatest potential to benefit from the digital logbook.

Several countries have now implemented strategies or enacted legislation to incorporate digital health interventions into reimbursement frameworks [25,26]. As a result, robust evidence from both RCTs and RWD is essential to support the value-based assessment of these interventions [25]. For the mySugr digital health intervention, the RCT [9] demonstrated efficacy in reducing diabetes distress—one of the most prevalent mental health challenges among people with diabetes [10,11,27]. This subsequent analysis contributes additional evidence on potential glycemic benefits, thereby strengthening the overall evidence base supporting the use of the mySugr digital diabetes logbook.

Future studies should explore the real-world use of the app by analyzing how engagement with specific app features predicts changes in glycemic outcomes. This would enable a more mechanistic understanding of which features—and at what dose or frequency—are most effective in achieving glycemic improvements [1]. In addition, it would be valuable to examine the real-world effects of a digital diabetes logbook on psychosocial outcomes, such as diabetes distress.

## Appendix

Multimedia Appendix 1 Additional analysis.

Multimedia Appendix 2 Number of participants achieving optimal glycemic control.

## Abbreviations

AACE: American Association of Clinical Endocrinologists
CGM: continuous glucose monitoring
eHbA1c: estimated glycated hemoglobin
GDM: gestational diabetes mellitus
HbA1c: glycated hemoglobin
RCT: randomized controlled trial
RWD: real-world data

## Data Availability

Anonymized data, without any demographic identifiers, underlying the results and analysis can be made available to researchers upon reasonable request to the corresponding author after publication. A data access agreement needs to be signed in advance.
