# Supplementary material for: Improvements in Glycemic Control With a Digital Diabetes Logbook: Secondary Analysis of a Randomized Controlled Trial Enriched by Observational, Real-World Data
Source: J Med Internet Res. 2025 Jun 30;27:e68933. doi: 10.2196/68933 (PMC12260466; doi:10.2196/68933)
Supplement: Multimedia Appendix 1 [file jmir_v27i1e68933_app1.docx]

**Supplementary Material:**

**Improvements in glycaemic control with a digital diabetes logbook: A secondary analysis of a randomised controlled trial enriched by observational, real-world data**

Authors:

Dominic Ehrmann (PhD)^1,2^, Bernhard Ruch (PhD)^3,4^, Michael Mitter (PhD)^3,4^, Johanna Kober (PhD)^3,4^, Norbert Hermanns (PhD)^1,2,5^, Vanessa Schäfer (PhD)^6^, Bernhard Kulzer* (PhD)^1,2,5^ & Stephan Silbermann* (MD)^4^

Affiliations:

1 Research Institute Diabetes Academy Mergentheim (FIDAM), Johann-Hammer-Str. 24, 97980 Bad Mergentheim, Germany

2 Department of Clinical Psychology and Psychotherapy, University of Bamberg, Markusplatz 3, 96047 Bamberg, Germany

3 mySugr GmbH Trattnerhof 1, 1010 Vienna, Austria

4 Roche Diabetes Care GmbH, Sandhofer Straße 116, 68305 Mannheim, Germany

5 Diabetes Clinic Mergentheim, Theodor-Klotzbuecher-Str. 12, 97980 Bad Mergentheim, Germany

6 Roche Diabetes Care Deutschland GmbH, Sandhofer Straße 116, 68305 Mannheim, Germany

Corresponding author:

Dr. Dominic Ehrmann, Research Institute Diabetes Academy Mergentheim (FIDAM), Johann-Hammer-Str. 24, 97980 Bad Mergentheim, Germany, +49 7931 96 192 42, ehrmann@fidam.de

* Stephan Silbermann and Bernhard Kulzer share last authorship

Supplementary Figure S1. Cohen’s D values for all features used in the propensity matching procedure between the RCT study cohort and the mySugr database.


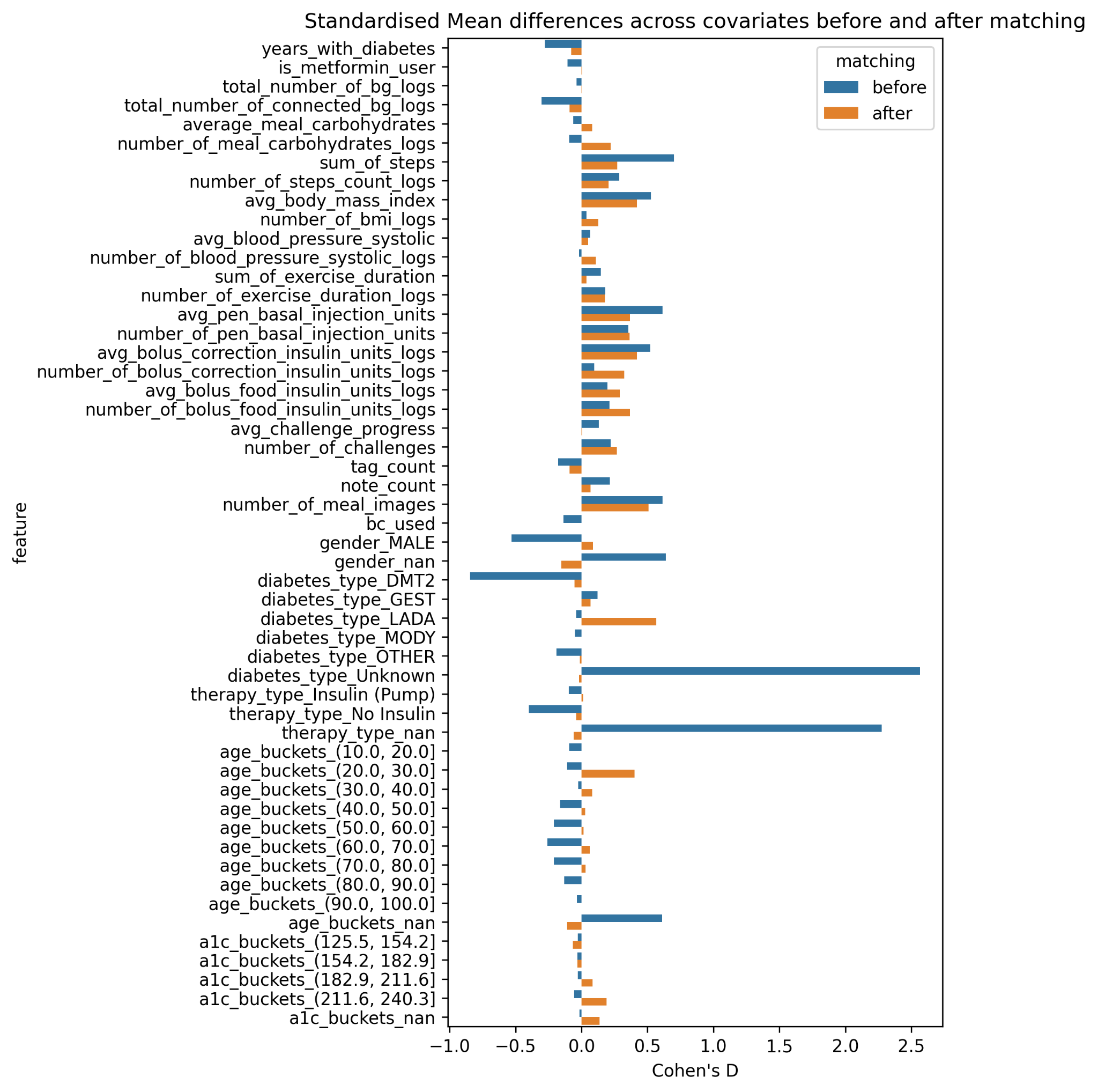


Supplementary Figure S2. Mean glucose levels before and during three months of app use in the total sample (A), in people with type 1 and type 2 diabetes (B), and stratified by baseline eHbA1c in type 1 diabetes and type 2 diabetes (C).

*A)*

*
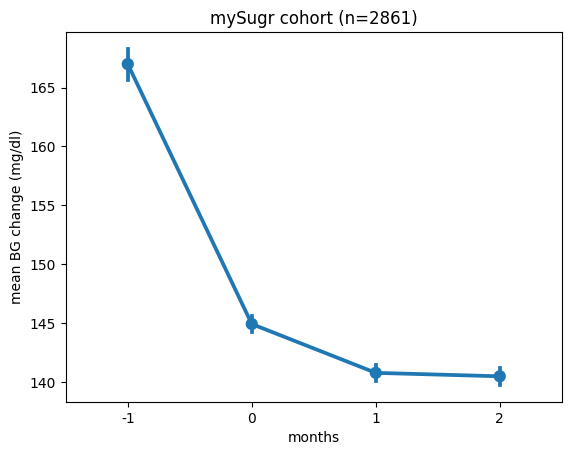
*

*B)*

*
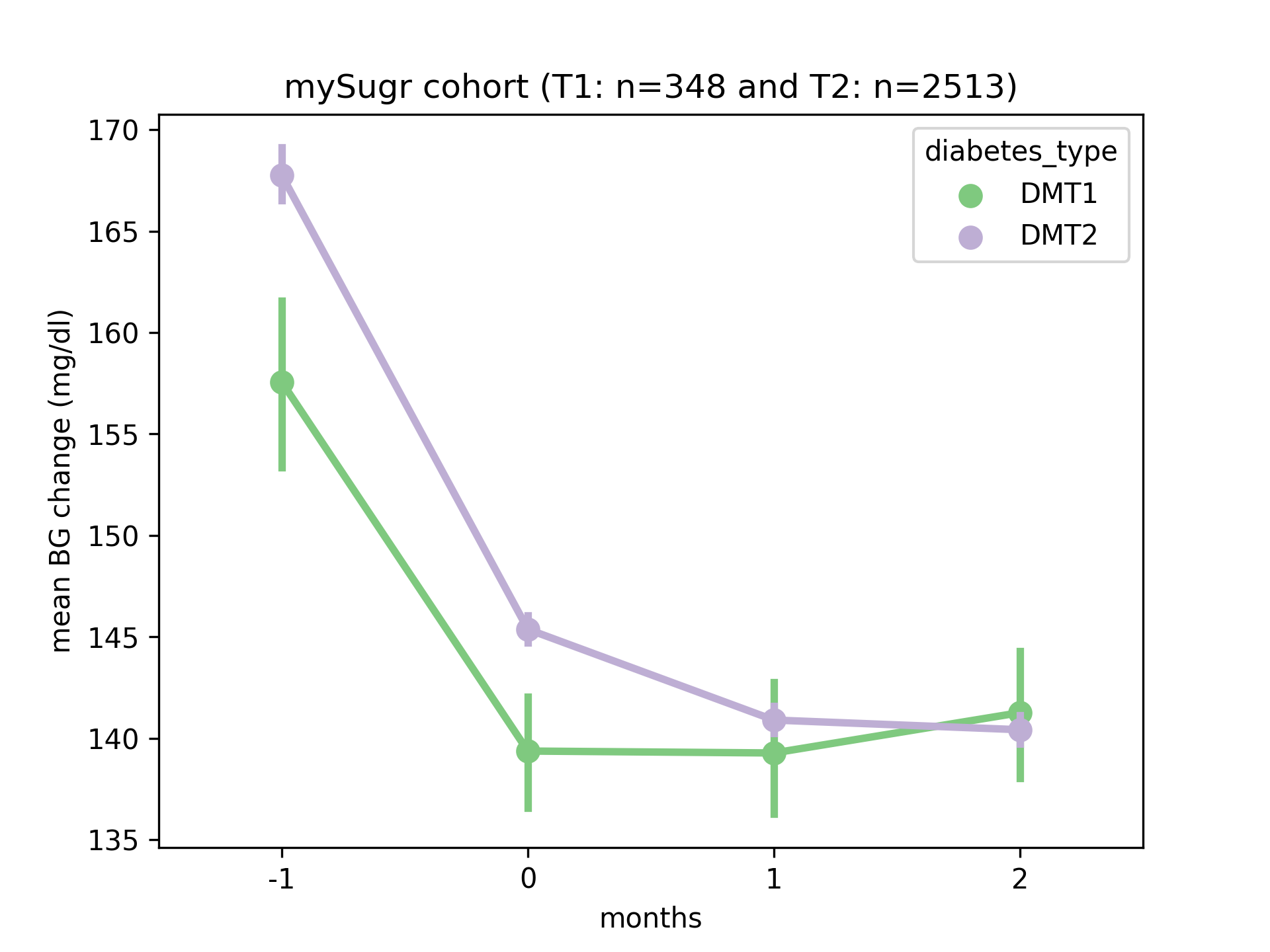
*

*C)*


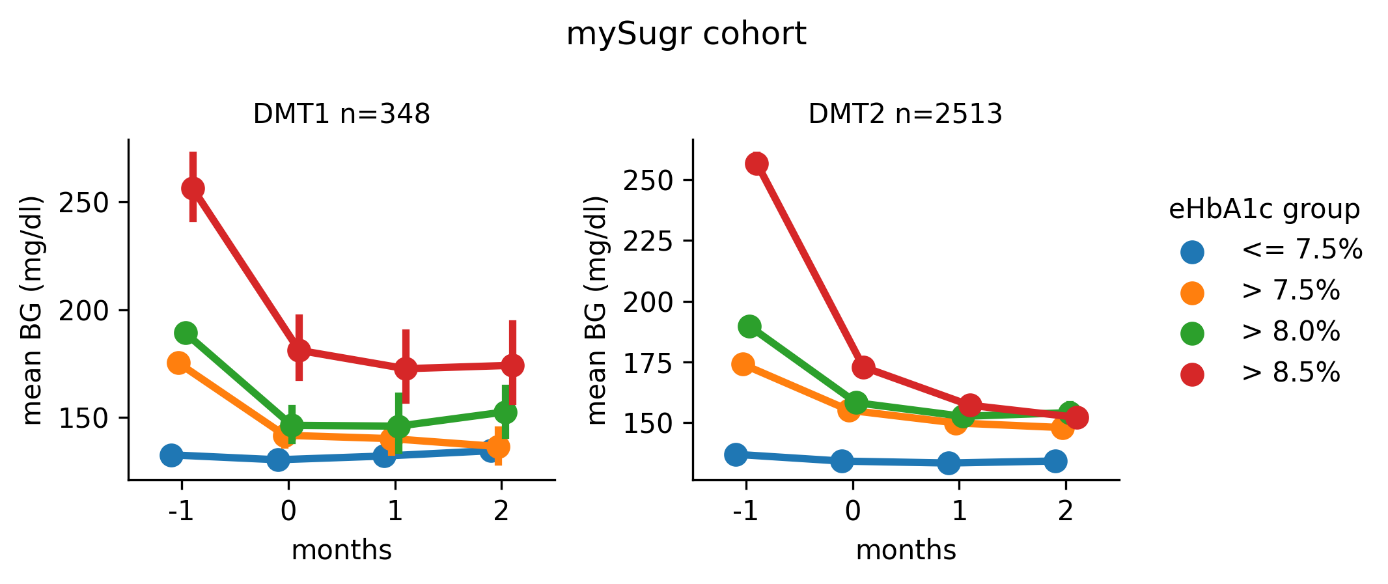


Supplementary Figure S3. Mean glucose levels before and during 12 months of app use in the total sample (A), in people with type 1 and type 2 diabetes (B), and stratified by baseline eHbA1c in type 1 diabetes and type 2 diabetes (C).

*A)*


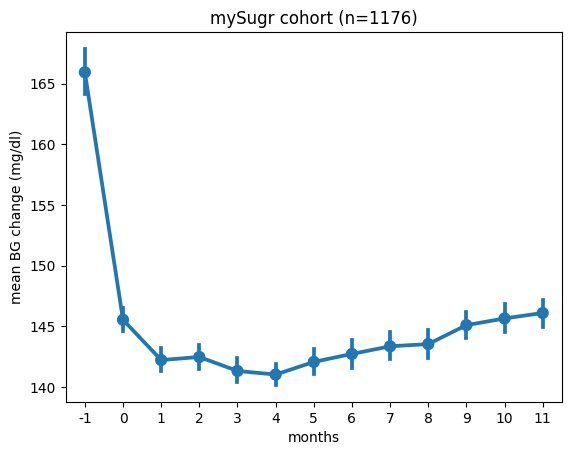


B)


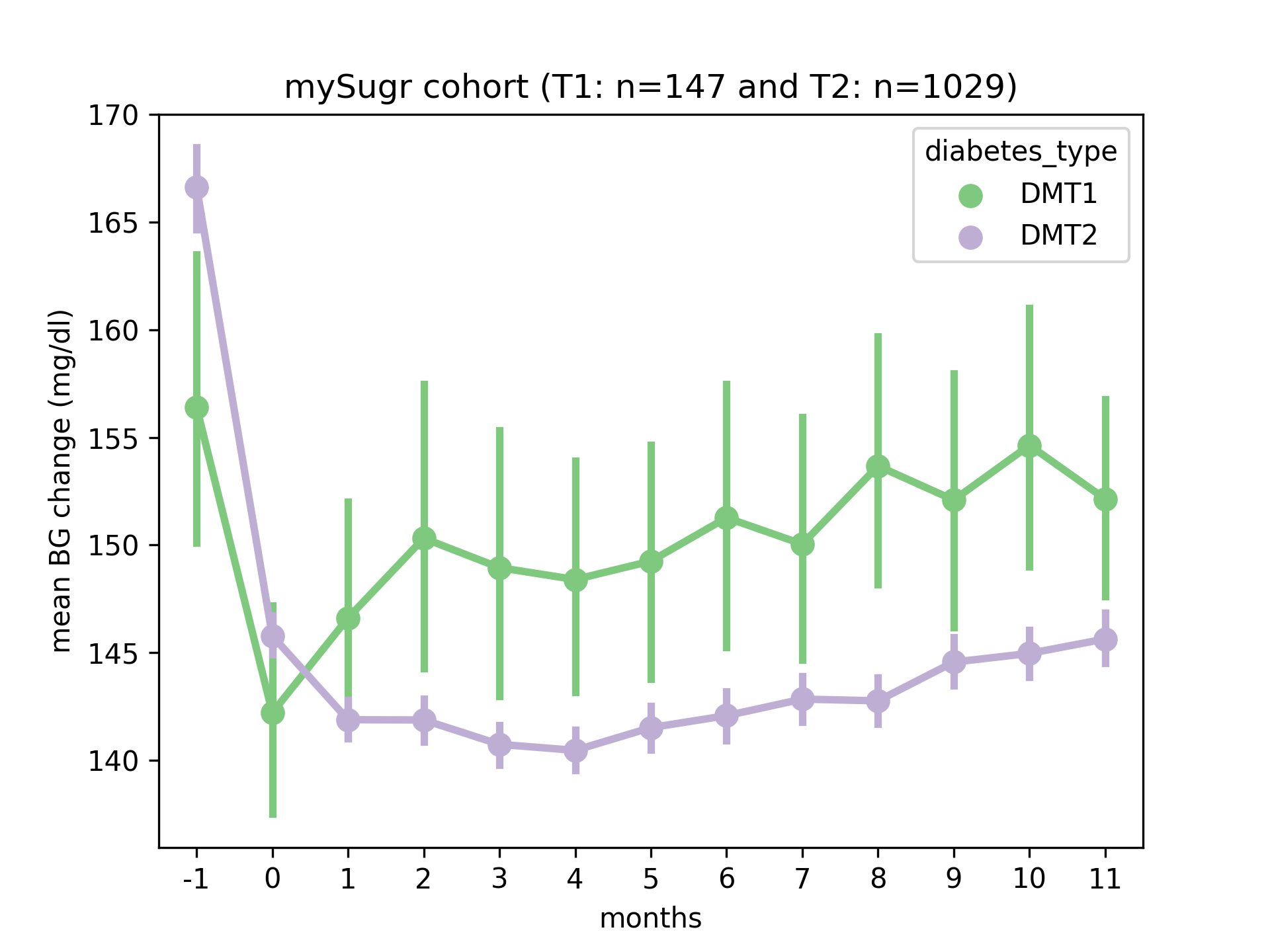


C)


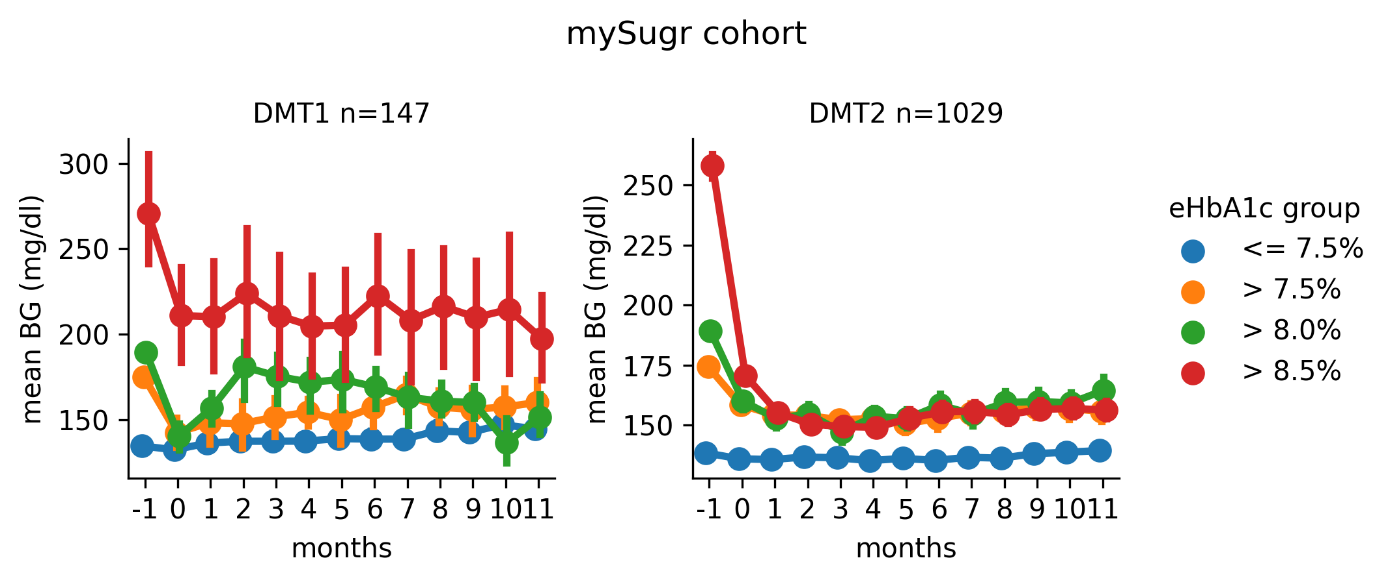


Supplementary Table S1. Reduction in mean blood glucose levels in mg/dl relative to baseline (before app use) for three months of app use in the total sample and for type 1 and type 2 diabetes (real-world data)

|  | Total sample (N = 2,861) | | Type 1 diabetes (n = 348) | | Type 2 diabetes (n = 2,513) | |
| --- | --- | --- | --- | --- | --- | --- |
|  | Mean (SD)  mg/dl | 95% CI | Mean (SD)  mg/dl | 95% CI | Mean (SD)  mg/dl | 95% CI |
| Month -1  Before app use | 0 | - | 0 | - | 0 | - |
| Month 0 | -22.1 (47.7) | -23.1 to -21.0 | -18.9 (41.6) | -21.6 to -14.8 | -22.4 (48.2) | -23.5 to -21.3 |
| Month 1 | -26.2 (55.5) | -27.4 to -25.1 | -18.3 (47.6) | -22.0 to -14.9 | -26.9 (56.1) | -28.1 to -25.4 |
| Month 2 | -26.5 (58.1) | -27.8 to -25.3 | -16.3 (49.7) | -20.6 to -12.4 | -27.3 (58.7) | -28.7 to -25.9 |

Supplementary Table S2. Reduction in mean blood glucose levels in mg/dl relative to baseline (before app use) for three months of app use in people with type 1 diabetes stratified by estimated baseline HbA1c (real-world data)

|  | eHbA1c ≤7.5% (n=223) | | eHbA1c 7.6-8.0% (n=39) | | eHbA1c 8.1-8.5% (n=22) | | eHbA1c >8.5% (n=64) | |
| --- | --- | --- | --- | --- | --- | --- | --- | --- |
|  | Mean (SD)  mg/dl | 95% CI | Mean (SD)  mg/dl | 95% CI | Mean (SD)  mg/dl | 95% CI | Mean (SD)  mg/dl | 95% CI |
| Month -1  Before app use | 0 | - | 0 | - | 0 | - | 0 | - |
| Month 0 | -2.3 (21.4) | -4.3 to -0.1 | -33.6 (17.1) | -37.6 to -29.6 | -42.8 (24.1) | -49.3 to -35.0 | -75.3 (73.6) | -91.6 to -58.8 |
| Month 1 | -0.5 (23.7) | -2.8 to 1.8 | -35.1 (25.8) | -41.4 to -29.0 | -43.2 (40.7) | -53.5 to -30.7 | -83.8 (81.6) | -104.1 to -66.1 |
| Month 2 | 1.9 (25.1) | -0.3 to 4.4 | -38.8 (30.5) | -46.4 to -31.9 | -36.6 (36.3) | -47.4 to -25.9 | -82.2 (87.7) | -102.0 to -61.8 |

Supplementary Table S3. Reduction in mean blood glucose levels in mg/dl relative to baseline (before app use) for three months of app use in people with type 2 diabetes stratified by estimated baseline HbA1c (real-world data)

|  | eHbA1c ≤7.5% (n=1,645) | | eHbA1c 7.6-8.0% (n=181) | | eHbA1c 8.1-8.5% (n=174) | | eHbA1c >8.5% (n=513) | |
| --- | --- | --- | --- | --- | --- | --- | --- | --- |
|  | Mean (SD)  mg/dl | 95% CI | Mean (SD)  mg/dl | 95% CI | Mean (SD)  mg/dl | 95% CI | Mean (SD)  mg/dl | 95% CI |
| Month -1  Before app use | 0 | - | 0 | - | 0 | - | 0 | - |
| Month 0 | -2.8 (16.9) | -3.3 to -2.3 | -19.0 (19.8) | -20.6 to -17.2 | -31.4 (26.7) | -33.6 to -29.1 | -83.9 (72.4) | -87.4 to -80.1 |
| Month 1 | -3.6 (20.9) | -4.2 to -3.0 | -24.2 (24.9) | -26.3 to -22.1 | -37.0 (30.2) | -39.6 to -34.4 | -99.6 (81.9) | -104.0 to -95.8 |
| Month 2 | -2.8 (22.3) | -3.4 to -2.1 | -26.1 (29.9) | -28.8 to -23.4 | -35.5 (38.5) | -38.7 to -32.4 | -104.6 (82.5) | -109.1 to -100.3 |

Supplementary Table S4. Reduction in mean blood glucose levels in mg/dl relative to baseline (before app use) for 12 months of app use in the total sample and for type 1 and type 2 diabetes (real-world data)

|  | Total sample (N = 1,176) | | Type 1 diabetes (n = 147) | | Type 2 diabetes (n = 1,029) | |
| --- | --- | --- | --- | --- | --- | --- |
|  | Mean (SD)  mg/dl | 95% CI | Mean (SD)  mg/dl | 95% CI | Mean (SD)  mg/dl | 95% CI |
| Month -1  Before app use | 0 | - | 0 | - | 0 | - |
| Month 0 | -20.4 (45.0) | -21.9 to -18.8 | -14.2 (39.5) | -19.8 to -9.3 | -20.9 (45.4) | -22.4 to -19.3 |
| Month 1 | -23.7 (52.5 | -25.6 to -21.9 | -9.8 (44.2) | -15.9 to -4.3 | -24.8 (52.9) | -26.8 to -22.9 |
| Month 2 | -23.5 (54.9) | -25.5 to -21.6 | -6.1 (47.0) | -12.4 to -0.5 | -24.8 (55.2) | -26.8 to -22.8 |
| Month 3 | -24.6 (55.0) | -26.5 to -22.8 | -7.5 (48.1) | -13.9 to -1.4 | -25.9 (55.3) | -28.0 to -23.9 |
| Month 4 | -24.9 (54.9) | -26.8 to -23.1 | -8.0 (48.4) | -14.6 to -2.0 | -26.2 (55.1) | -28.2 to -24.3 |
| Month 5 | -23.9 (54.9) | -25.8 to -22.1 | -7.1 (48.1) | -13.6 to -1.3 | -25.1 (55.2) | -27.1 to -23.4 |
| Month 6 | -23.2 (54.8) | -25.1 to -21.4 | -5.1 (47.5) | -11.4 to 0.7 | -24.6 (55.1) | -26.6 to -22.8 |
| Month 7 | -22.6 (55.5) | -24.5 to -20.7 | -6.4 (48.8) | -13.0 to -0.5 | -23.8 (55.8) | -25.8 to -21.9 |
| Month 8 | -22.4 (55.8) | -24.4 to -20.5 | -2.7 (48.3) | -10.0 to 3.3 | -23.9 (56.0) | -26.0 to -21.8 |
| Month 9 | -20.8 (55.8) | -22.7 to -19.0 | -4.3 (50.8) | -11.5 to 2.3 | -22.1 (56.0) | -24.1 to -20.1 |
| Month 10 | -20.3 (56.2) | -22.3 to -18.4 | -1.8 (56.3) | -9.5 to 5.2 | -21.7 (56.0) | -23.8 to -19.7 |
| Month 11 | -19.8 (57.3) | -21.8 to -17.9 | -4.3 (49.8) | -11.1 to 2.1 | -21.0 (57.7) | -23.1 to -19.0 |

Supplementary Table S5. Reduction in mean blood glucose levels in mg/dl relative to baseline (before app use) for 12 months of app use in people with type 1 diabetes stratified by estimated baseline HbA1c (real-world data)

|  | eHbA1c ≤7.5% (n=102) | | eHbA1c 7.6-8.0% (n= 15) | | eHbA1c 8.1-8.5% (n= 6) | | eHbA1c >8.5% (n= 24) | |
| --- | --- | --- | --- | --- | --- | --- | --- | --- |
|  | Mean (SD)  mg/dl | 95% CI | Mean (SD)  mg/dl | 95% CI | Mean (SD)  mg/dl | 95% CI | Mean (SD)  mg/dl | 95% CI |
| Month -1  Before app use | 0 | - | 0 | - | 0 | - | 0 | - |
| Month 0 | -2.3 (17.4) | -4.9 to 0.6 | -32.7 (19.0) | -40.6 to -24.5 | -49.2 (17.9) | -57.7 to -39.8 | -59.7 (91.1) | -97.6 to -27.9 |
| Month 1 | 1.6 (23.8) | -1.9 to 5.3 | -26.9 (26.8) | -38.0 to -15.8 | -32.7 (19.1) | -42.4 to -23.1 | -60.4 (99.8) | -101.9 to -24.3 |
| Month 2 | 2.7 (26.0) | -1.2 to 6.8 | -27.4 (30.8) | -40.6 to -15.1 | -8.5 (33.6) | -26.0 to 6.2 | -46.8 (111.8) | -93.2 to -5.7 |
| Month 3 | 2.7 (30.3) | -1.7 to 7.8 | -23.4 (24.9) | -34.1 to -13.2 | -14.1 (28.4) | -28.8 to -1.0 | -59.8 (105.2) | -101.8 to -22.1 |
| Month 4 | 2.8 (28.5) | -1.6 to 7.1 | -20.7 (19.0) | -28.6 to -12.9 | -17.5 (30.6) | -34.6 to -4.7 | -65.9 (107.5) | -109.5 to -27.7 |
| Month 5 | 4.2 (26.7) | 0.4 to 8.6 | -25.0 (31.0) | -37.8 to -13.2 | -15.9 (32.7) | -32.2 to -0.8 | -65.2 (105.8) | -108.8 to -28.7 |
| Month 6 | 3.9 (27.0) | 0.01 to 8.2 | -17.7 (24.8) | -28.1 to -7.2 | -20.3 (23.7) | -32.1 to -9.7 | -48.0 (114.3) | -94.5 to -7.0 |
| Month 7 | 4.0 (25.4) | 0.3 to 8.0 | -10.5 (24.5) | -21.2 to -1.0 | -26.4 (28.3) | -41.0 to -13.6 | -62.4 (115.3) | -109.3 to -18.4 |
| Month 8 | 8.8 (29.6) | 4.4 to 13.7 | -17.7 (22.3) | -27.3 to -8.3 | -28.5 (19.2) | -36.5 to -16.9 | -54.4 (107.1) | -100.2 to -16.5 |
| Month 9 | 7.8 (30.7) | 3.5 to 13.1 | -19.1 (31.7) | -32.6 to -6.2 | -29.4 (18.8) | -38.3 to -19.3 | -60.8 (110.7) | -104.3 to -21.8 |
| Month 10 | 12.4 (37.9) | 6.9 to 18.6 | -17.6 (26.2) | -29.1 to -7.1 | -52.9 (27.9) | -65.1 to -38.7 | -56.1 (114.5) | -105.3 to -13.0 |
| Month 11 | 9.9 (30.6) | 5.5 to 14.5 | -15.0 (30.0) | -28.1 to -3.6 | -38.0 (24.2) | -48.8 to -24.2 | -73.2 (95.6) | -112.8 to -42.0 |

Supplementary Table S6. Reduction in mean blood glucose levels in mg/dl relative to baseline (before app use) for 12 months of app use in people with type 2 diabetes stratified by estimated baseline HbA1c (real-world data)

|  | eHbA1c ≤7.5% (n=696) | | eHbA1c 7.6-8.0% (n= 73) | | eHbA1c 8.1-8.5% (n= 65) | | eHbA1c >8.5% (n= 195) | |
| --- | --- | --- | --- | --- | --- | --- | --- | --- |
|  | Mean (SD)  mg/dl | 95% CI | Mean (SD)  mg/dl | 95% CI | Mean (SD)  mg/dl | 95% CI | Mean (SD)  mg/dl | 95% CI |
| Month -1  Before app use | 0 | - | 0 | - | 0 | - | 0 | - |
| Month 0 | -2.4 (15.6) | -3.1 to -1.8 | -15.9 (17.2) | -18.1 to -13.5 | -29.3 (26.5) | -32.5 to -26.1 | -87.4 (66.3) | -93.3 to -81.7 |
| Month 1 | -2.8 (17.7) | -3.5 to -2.0 | -20.1 (21.1) | -22.7 to -17.0 | -36.5 (29.6) | -40.2 to -32.6 | -103.0 (76.8) | -109.2 to -96.6 |
| Month 2 | -1.7 (18.9) | -2.5 to -0.9 | -20.2 (21.7) | -23.2 to -17.0 | -34.7 (33.0) | -38.8 to -30.6 | -107.7 (77.9) | -113.7 to -101.3 |
| Month 3 | -2.0 (19.6) | -2.9 to -1.2 | -22.1 (23.3) | -25.1 to -18.8 | -42.2 (31.4) | -46.2 to -38.1 | -108.7 (76.4) | -115.2 to -102.0 |
| Month 4 | -3.2 (19.7) | -4.1 to -2.4 | -21.6 (25.6) | -24.8 to -18.1 | -35.6 (30.7) | -39.5 to -32.0 | -109.0 (77.0) | -115.4 to -102.7 |
| Month 5 | -2.3 (20.7) | -3.2 to -1.3 | -23.8 (26.6) | -27.3 to -20.1 | -36.6 (32.3) | -40.8 to -32.6 | -105.2 (78.8) | -111.7 to -98.7 |
| Month 6 | -3.1 (21.3) | -4.0 to -2.1 | -21.6 (31.8) | -26.1 to -16.9 | -30.9 (36.9) | -35.6 to -26.3 | -102.6 (80.0) | -109.3 to -96.0 |
| Month 7 | -1.8 (21.5) | -2.6 to -0.8 | -19.3 (31.3) | -23.6 to -14.9 | -34.7 (37.0) | -39.7 to -30.2 | -102.3 (81.3) | -108.9 to -95.5 |
| Month 8 | -2.2 (22.7) | -3.2 to -1.2 | -18.5 (30.3) | -22.4 to -14.2 | -29.9 (40.4) | -34.9 to -25.2 | -103.7 (79.5) | -110.3 to -97.1 |
| Month 9 | -0.3 (21.6) | -1.2 to 0.7 | -17.0 (32.6) | -21.4 to -12.3 | -29.5 (39.0) | -34.3 to -24.7 | -101.6 (80.7) | -108.7 to -95.0 |
| Month 10 | 0.3 (21.3) | -0.6 to 1.3 | -17.4 (33.3) | -21.9 to -12.7 | -30.1 (35.9) | -34.4 to -25.4 | -101.1 (81.3) | -107.6 to -94.1 |
| Month 11 | 1.0 (25.3) | -0.1 to 2.1 | -18.6 (30.7) | -22.5 to -14.4 | -24.8 (42.2) | -30.1 to -19.5 | -101.9 (81.4) | -109.0 to -94.5 |
