# Supplementary material for: Improvements in Glycemic Control With a Digital Diabetes Logbook: Secondary Analysis of a Randomized Controlled Trial Enriched by Observational, Real-World Data
Source: J Med Internet Res. 2025 Jun 30;27:e68933. doi: 10.2196/68933 (PMC12260466; doi:10.2196/68933)
Supplement: Multimedia Appendix 2 [file jmir_v27i1e68933_app2.docx]

| Type 1 & type 2 diabetes only | Randomized controlled trial | | Real-world cohort |
| --- | --- | --- | --- |
|  | Control group | Intervention group | Existing app users |
| Intention-to-treat population: 3 months after baseline^a^ | | | 3 months after baseline^b^ |
| Follow-up HbA_1c_ > 6.5% | 86 (73.5%) | 151 (67.1%) | 1191 (41.6%) |
| Follow-up HbA_1c_ ≤ 6.5% | 31 (26.5%) | 74 (32.9%) | 1670 (58.4%) |
| **Per-protocol population: 3 months after baseline^a^** | | | **12 months after baseline^b^** |
| Follow-up HbA_1c_ > 6.5% | 76 (74.5%) | 124 (67.8%) | 529 (45.0%) |
| Follow-up HbA_1c_ ≤ 6.5% | 26 (25.5%) | 59 (32.2%) | 647 (55.0%) |
| ^a^ Laboratory-measured HbA_1c_.  ^b^Estimated HbA_1c_ based on the mean blood glucose (see formula by Nathan et al [14]). | | | |
